# Explorative Analysis of Wuhan Intra-Urban Human Mobility Using Social Media Check-In Data

**DOI:** 10.1371/journal.pone.0135286

**Published:** 2015-08-19

**Authors:** Lin Li, Lei Yang, Haihong Zhu, Rongrong Dai

**Affiliations:** School of Resources and Environmental Science, Wuhan University, Wuhan, China; Oswaldo Cruz Foundation, BRAZIL

## Abstract

Social media check-in data as a geo-tagged information source have been used for revealing spatio-temporal patterns in the field of social and urban study, such as human behavior or public issues. This paper investigates a case study and presents a new method of representing the mobility of people within a city from check-in data. By dividing the data in a temporal sequence, this study examines the overall mobility in the case study city through the gradient/difference of population density with a series of time after computing the population density from the check-in data using an incorporated Thiessen polygon method. By classifying check-in data with their geo-tags into several groups according to travel purposes, and partitioning the data according to administrative district boundaries, various moving patterns for those travel purposes in those administrative districts are identified by scrutinizing a series of spatial geometries of a weighted standard deviational ellipse (WSDE). Through deep analyses of those data by the adopted approaches, the general pattern of mobility in the case city, such as people moving to the central urban area from the suburb from 4 am to 8 am, is ascertained, and different characteristics of movement in those districts are also depicted. Furthermore, it can tell that in which district less movement is likely for a certain purpose (e.g., for dinner or entertainment). This study has demonstrated the availability of the proposed methodology and check-in data for investigating intra-urban human mobility.

## Introduction

Social media, which takes on many different forms including microblogs, social networks and photo sharing, is a very large source of information for examining spatio-temporal phenomena or human behavior in social and urban domains [1–5]. Social media is a new type of sensor, capturing extensive and up-to-date informative features about social, economic, geographic, demographic and human behavior in a city, and brings new perspectives to the study of social and urban regularities. The study of human mobility is of significance because the general patterns of human mobility are expected to be better understood for a long period in such areas as sociology and geography [6].

In general, modeling human movement in the inter-city or intra-city is tightly associated with urban models or is a type of urban modeling itself. As early as 1946, G. K. Zipf [7] proposed the gravity model for simulating inter-city human mobility. Because the model contains undetermined parameters such as the evaluation indicators of cities and the travel distance defined in the damping function, it could not be applied when travel survey data are lacking. To solve this problem, F. Simini [8] proposed the radiation model, which is parameter-free and has a good performance in inter-urban human mobility prediction. The rank-based movement model [9], the generalized potential model [10], and the intervening opportunities model [11] are additional models used to simulate and predict human mobility patterns on different levels. Intra-urban human mobility has shown its great importance in traffic accessibility, location-based services, and urban planning [12–14], and has been explored by many studies for its regularity and predictability [2, 8, 15, 16]. These models all operate from multiple perspectives and take into account such factors as geography, population, and social economy. Some trends and intensities of human mobility are revealed by means of mathematical deduction from empirical data sets.

Due to the growing popularity of location-based services and geo-social networks, users communicate more and more private location traces to service providers, as well as explicit spatio-temporal data, often called “check-ins”, about their presence in specific spots or venues at given times [17]. Information about time and location as well as photos is included in a check-in record. We can acquire when and where the check-in activities happened and extract the footprints of a large number of individuals [1]. Check-in data as geo-tagged information sources has been used for revealing some spatio-temporal regularity in urban areas such as identifying commercial centers [18], detecting local events [19] and determining population distribution [20].

For mining patterns of human mobility from check-in data, some studies substantially address that topic. Liu, Y. et al. [1] extract inter-urban movements in China from a check-in data set to analyze the underlying patterns of trips and spatial interactions and construct a spatial network where the edge weights denote the interaction strengths. They find that the communities detected from the network are spatially cohesive and roughly consistent with province boundaries. The approach links patterns at the collective level of spatial interactions versus the individual level of human movements from mobile phone or taxi data sets. Noulas, A. et al. [9] study urban mobility patterns in several metropolitan cities, which verify variations in human movement caused by different distributions of places across different urban environments, and indicate that the probability of transiting from one place to another is inversely proportional to a power of their rank. Wu, L. et al. [21] construct a temporal transition probability matrix to represent the transition probability of travel demands during a time interval, and adopt the mechanism of an agent-based model, which combines activity-based analysis with a movement-based approach. Most existing human mobility models capture real human movement to some degree, but assume continuous and homogeneous space [22]. The gap between assumption and reality could be bridged by some devised spatialization approach in processing check-in data.

By far, all studies of human mobility based on check-in data are a type of case study revealing spatio-temporal features of human mobility. The intra-urban movement of individuals is affected by a number of factors, such as urban form, mode of transportation, transportation networks and socio-economic status [23–26]. Movement patterns dependent on a specific city could not be applied to other cities or urban areas. Thus, the uniqueness of human mobility to the city requires investigating a city individually, and the revealed patterns or features are irreplaceable for the city in urban planning and decision-making.

This study takes Wuhan, China, as the case study city and explores the spatio-temporal pattern of intra-urban human mobility using check-in data collected over a year-long period (from March 2013 to March 2014). The contributions of this paper are that the general pattern of the movement of people in a suburb-urban area in the city is depicted and that spatio-temporal characteristics of the movement of people for some given travel purposes in districts are revealed from the check-in data by the methodology proposed in this paper.

The remainder of this paper is structured as follows. Section 2 provides a brief introduction to the case study city and the spatio-temporal characteristics of the check-in data. The general temporal pattern of movement is then depicted according to classified travel-demands. Section 3 introduces a methodology of deriving population density from check-in data by incorporating Thiessen polygons and two-layer grids into a spatialization of discrete points, followed by presenting the general spatio-temporal pattern of human mobility between suburban and urban environments. More detailed spatio-temporal characteristics of movement for travel purposes in districts are presented in Section 5 from a series of geometric features of weighted standard ellipses depicted for each travel demand in every district. Finally, some conclusions are drawn from the exploration and discussion.

## Check-In Data in Wuhan

### Geography of the case city: Wuhan

**Fig 1:**
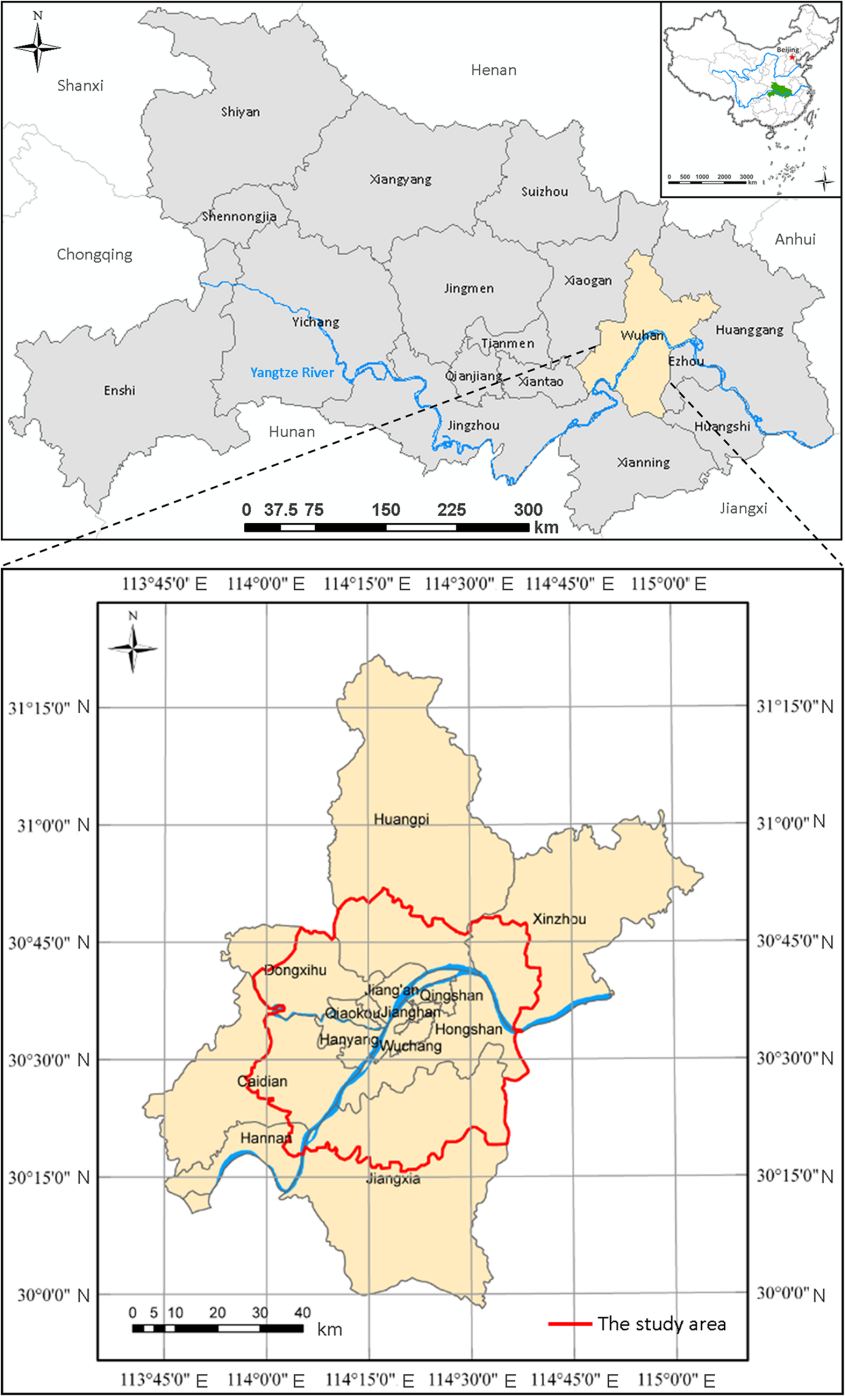
The geographical location of the case study city: Wuhan (the red line indicates the case study area).

Wuhan (29°58′–31°22′N, 113°41′–115°05′E), the capital of Hubei Province, is located in central China and is crossed by the Yangtze River, the longest river in China (Fig 1). Its main urban area is covered by seven urban districts (Jianghan, Jiang’an, Qiaokou, Hanyang, Wuchang, Hongshan and Qingshan). There is a population of approximately 10.22 million (as of year-end 2013) in the whole administrative unit covering an area of 8494 km^2^, among which water area accounts for about one quarter. Because of its favored geographical location in the inland of China, Wuhan has become a major comprehensive transportation hub. Considering the majority of contributions to the human activities of the city, the main urban area of Wuhan City plotted by city planners (in the bottom of Fig 1) is investigated as the extent of the intra-urban area in this study.

### Check-in data in the case study area

A check-in record contains some related information about activity of an individual when and where the check-in happens. Because check-in is a behavior of a user’s intention in his/her daily life, and check-in spots are allocated in different places, a set of check-in records can show a pattern of people’s mobility in a given area.

Check-in records in the period from March 2013 to March 2014 from the social media Sina Visitor System are collected for this city. Because of free Check-in behavior, some unavailable records due to factors such as the inconsistency of locations, and some redundancy, as well as records outside of the study area, will be removed from the data set through spatialization or geographic rectification of the records. Eventually, 770522 micro-blogging check-in records are kept, with 312190 users and 173114 venues/spots **(S1 and S2 Files)**.

### Travel demands based on check-in data

**Fig 2:**
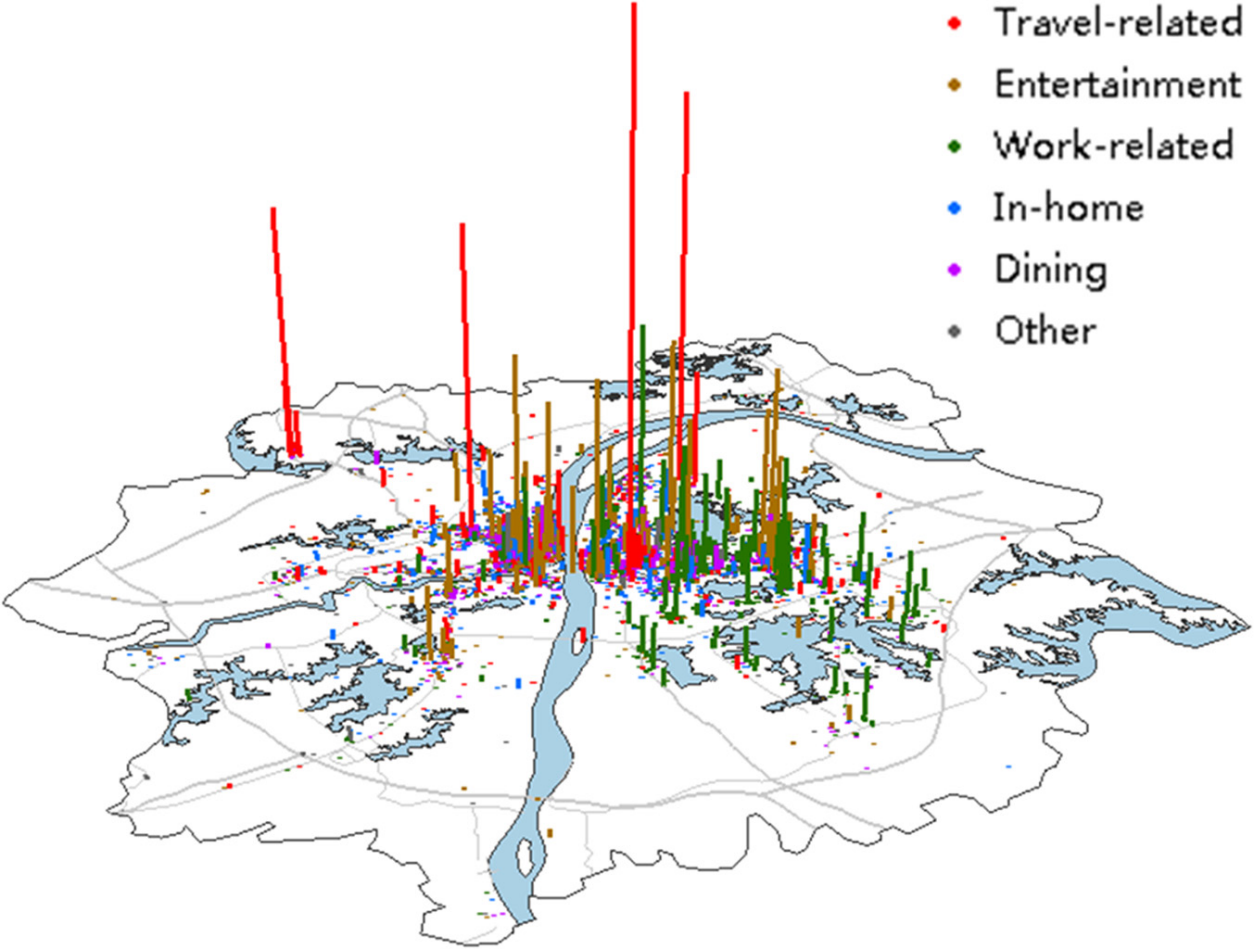
Spatial distribution of check-ins for different travel demands.

Regarding the movement of people in a city, check-in data have the merit of indicating the purpose of individual travel with the demand-tags associated with check-in activities over other data enabled for GPS (such as taxi trajectory data or mobile call records) [21]. The need to participate in activities generates travel demands [27–28], and thus detailed activity information for some purposes is very helpful for studying human travel behaviors, traffic engineering, and urban planning [29]. Decomposing check-in data into a series of activities can significantly improve our understanding of human mobility. Demand-tags are fitting to classify the activities for travel purposes. Following existing studies [14, 30, 31, 32] on classification of travel demands, and studies [33–38] on inferring the actual activities of individuals from trajectories based on POI data, the travel demands depending on the associated demand-tags are grouped into six categories: Travel-related, Entertainment, Work-related, In-home, Dining and Other. Each category of the check-in activity suggests a type of movement in the urban area, which characterizes intra-urban human mobility. Fig 2 shows the spatial distribution of check-ins in terms of the six travel demands, where the height of the bars indicates the number of check-in users. Additionally, check-in data are also divided in a series of 2-hour intervals of diurnal spatio-temporal data sets in a 24-hour cycle.

### General temporal features of the check-in data

**Fig 3:**
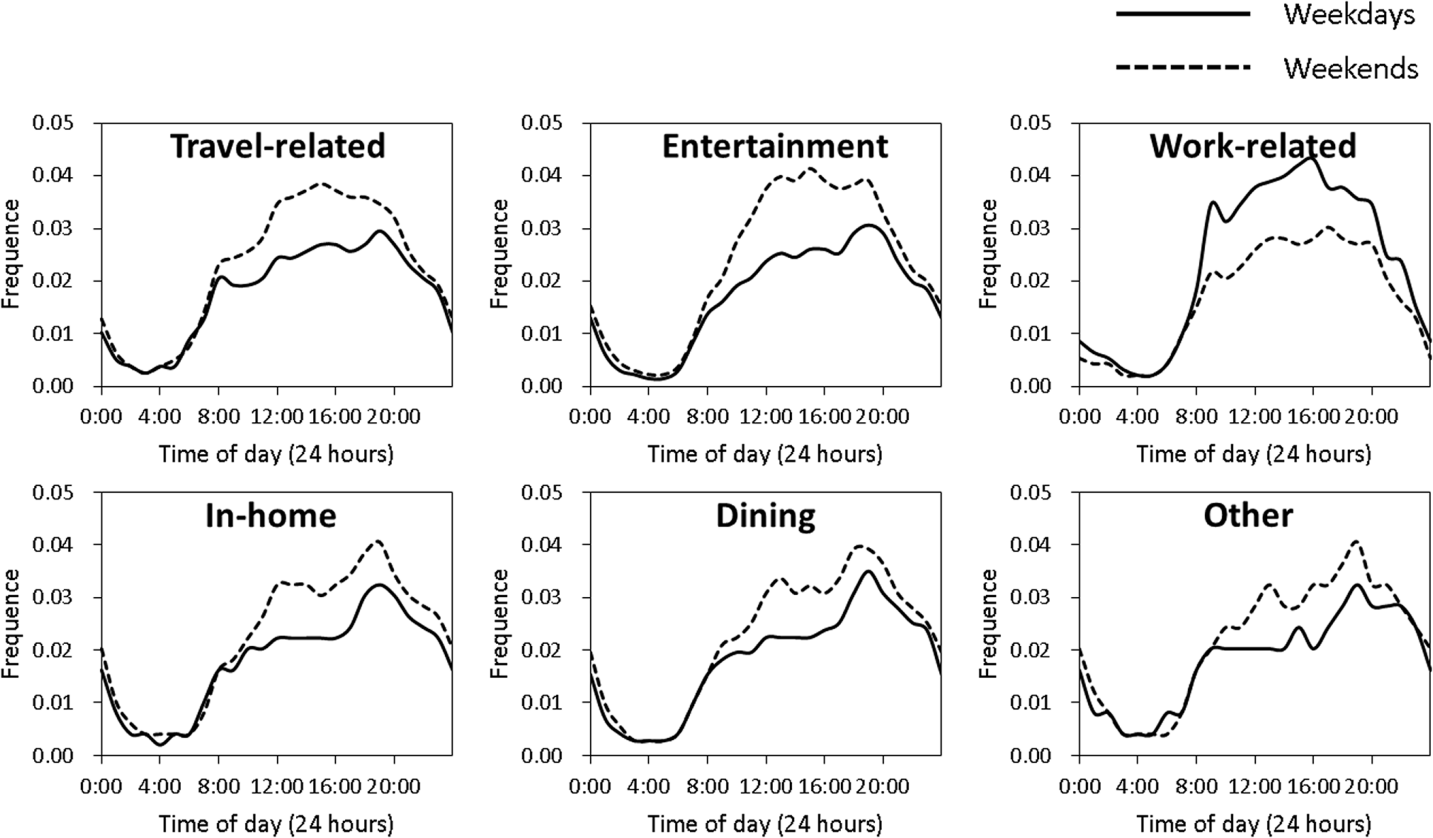
Diurnal temporal distributions of different demand-tagged trips.

Considering the intention and temporal distribution of checking-in activities, diurnal temporal distribution characteristics are depicted in Fig 3. These charts show that travels for Work-related are more active in weekdays, whereas travels for other purposes are more active in weekends. In terms of the characteristic shapes of the curves, travels for Transportation, Entertainment and Work-related exhibit inverted-U curves from 6 am to midnight. In weekdays, all travels except Work-related have their peak near 8 pm, meaning that more movements occur at that time. Those curves demonstrate the overall temporal characteristics of activities for different travel demands.

## Derivation of Population Density from Check-In Data

### Estimations of population density

Human mobility within an urban area can be regarded as a spatio-temporal phenomenon of the change of population density. Variation of checking-in activity embedded in space depicts the dynamics of population density. Thus, population density can be calculated from the intensity of checking-in activity. There are many studies of the calculation of population density [39–44]. In terms of implementation, two types of approaches are used for estimating population density distribution: areal interpolation and surface modeling. Areal interpolation refers to the process of transferring data from one set of areas to another [41], and it is optional whether ancillary information is used [42]. Population surface modeling uses asymmetric mapping and incorporates areal weighting and empirical sampling techniques to create a population map on a regular grid system, where each grid cell represents an estimate of the number of people for that individual cell [39, 43]. The division of the space is usually performed according to natural units or regular grids. One of the typical challenges in dividing the space is that it is difficult to acquire the actual boundary data of the unit cells because the boundary is designated for other purposes such as administration or planning.

### Calculation of population density based on Thiessen polygons

**Fig 4:**
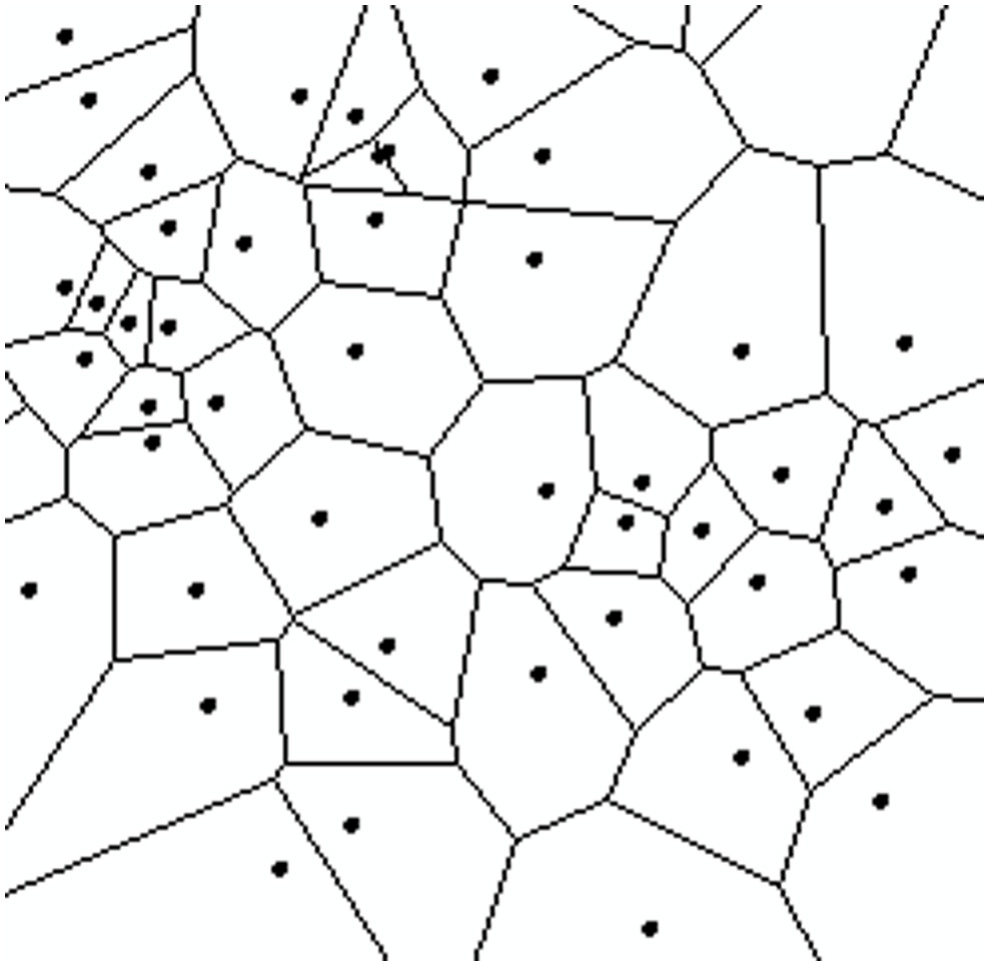
Thiessen polygon network constructed from discrete points.

A check-in spot can be perceived as an observation point representing accumulated population. Thiessen polygons appear to be a suitable modeling tool [45] and are a useful tool for calculating spatial distributions from discrete points (e.g., calculation of rain gauge density [46], transit traffic analysis [47] and urban area hotspots detection [48]), as shown in Fig 4.

Let *P* = {*P*_1_, *P*_2_, …, *P*_*n*_} denote the dataset of check-ins, where *n* ∈ [3, ∞), and let *d*(*P*_*i*_, *P*_*j*_) denote the Euclidean distance between *P*_*i*_ and *P*_*j*_. The cellular network of the check-ins made up of distributional Thiessen polygons (denoted by *V*_*i*_) can be created based on location data of the check-in places. At any point in this divisional space, *V*_*i*_ can be described by

$$V_{i}=\left\{p\in \left\{d(p,P_{i})\leq d(p,P_{j})\right\},\;i,j=1,2,\ldots ,n;\;i\neq j\right\}$$ (1)

Check-in places can be interpreted as centers of spatial objects and centers of population concentration, and their corresponding Thiessen polygons can be interpreted as the scope of their spatial influence, with population distribution being regarded as homogeneous within that scope. Consequently, the initial population density of check-ins at *P* in time period *T* based on Thiessen polygons is represented as

$$D_{P}^{T}=\frac{POP_{P}}{AREA_{V_{P}}}$$ (2)

Given that there is a layer of excluded areas for which travel is unlikely or impossible, such as areas of water, we set a population density threshold (denoted by *D*_th_) according to the relationship between the density of check-in points and the population density. Hence, the population density of *P* is denoted by

$$D_{P}=D_{P}^{T}+D_{\text{th}}$$ (3)

**Fig 5:**
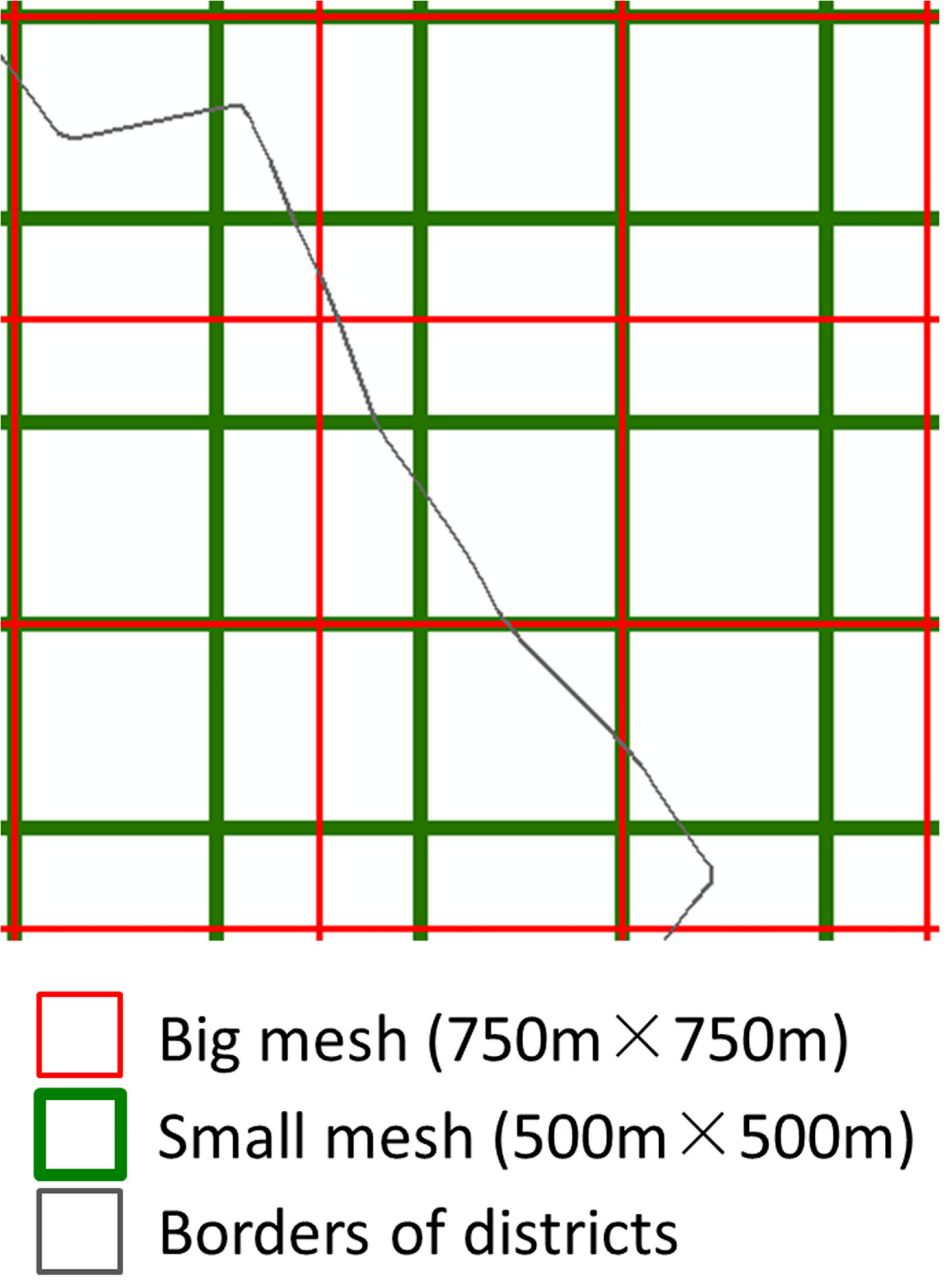
Superimposed grids of different size.

**Fig 6:**
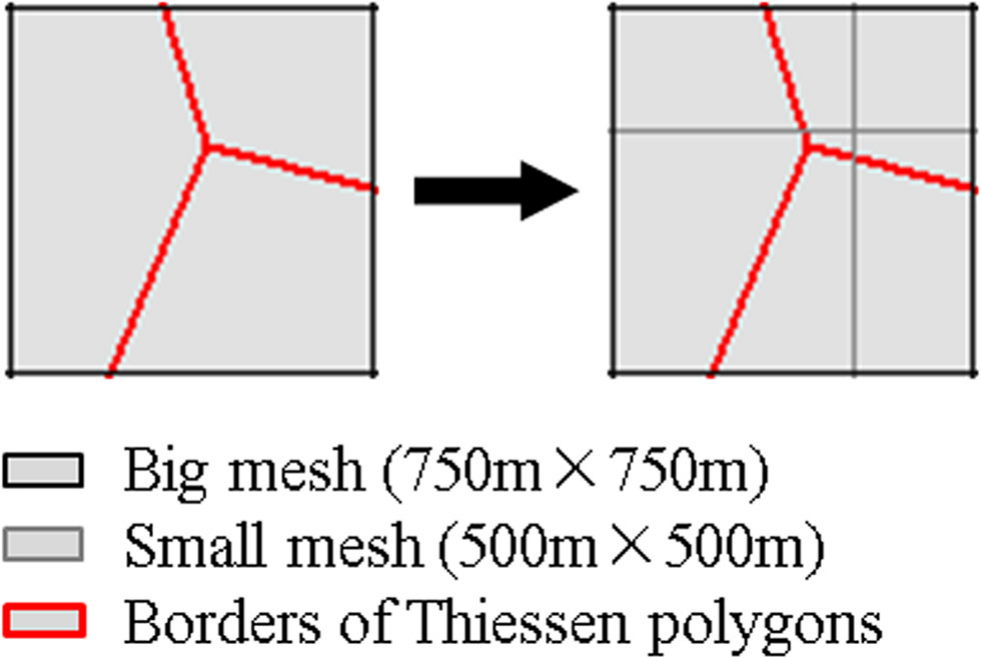
Area weighting interpolation.

Grid cell areal weighting interpolation (GCAWI) is one of the methods for spatializing population density [44]. Its core concepts are areal weighting interpolation and neighborhood averaging. To overcome the limitations regarding gradualness and continuity in the computation of population density, grids of different sizes (750m × 750m and 500m × 500m, as shown in Fig 5) are superimposed onto the network space divided according to Thiessen polygons. As a result, polygons composed of common edges shared by two Thiessen polygons are divided into several sub-polygons, as shown in Fig 6 Weighting by the areas of these sub-polygons, the final weighted population density is calculated by

$$D=\frac{\sum_{i=1}^{n}D_{i}\times AREA_{i}}{\sum_{i=1}^{n}AREA_{i}}\;(i=1,2,\ldots ,n)$$ (4)

This approach mitigates the mutant lines in population density distributions calculated by Thiessen polygons and relieves the discontinuous terraced boundaries so that the continuous spatial characteristics of human mobility could be better presented.

**Fig 7:**
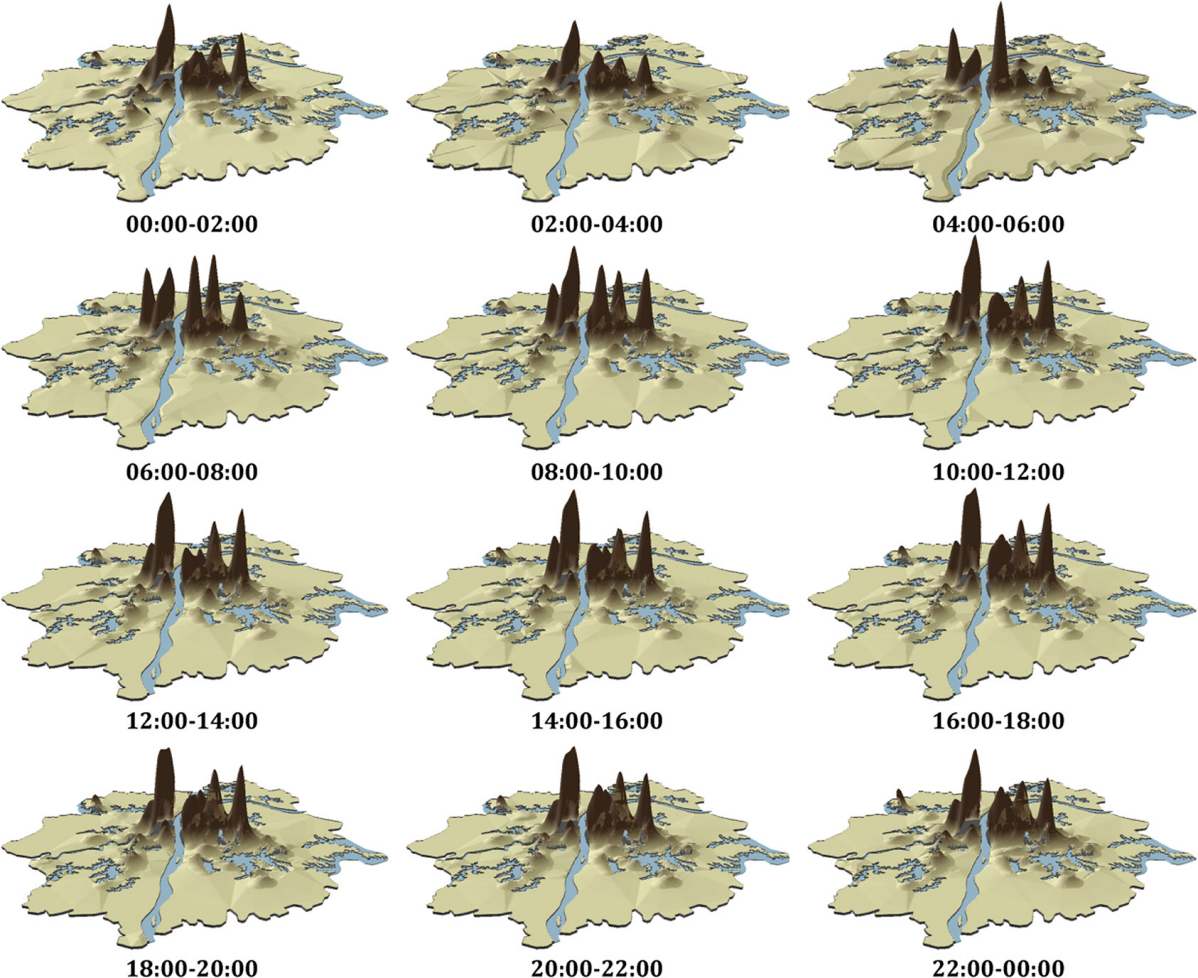
Spatio-temporal distribution of population density.

After calibration of Thiessen polygons and the GCAWI algorithm by allocating regional population (from statistical yearbook data) to this study area, twelve time-interval sets of population density are calculated as shown in Fig 7. When overlaying the city map with those density sets, it is found that the distribution of several areas with clearly high population densities coincided with the distribution of larger commercial centers in Wuhan, such as the Jianghan Road commercial center and the Jiangtan commercial center. Those data also indicate a large amount of population flow during the 6 am to 10 am period.

## Human Mobility in Suburban-Urban Areas from Density Change

If a point *p* in the plan of the study area has some population density *f* at some time *t*_0_, according to mathematic concepts, the gradient of population at *p* for time-interval Δ*t* = *t*_1_−*t*_0_ is denoted by

$$Grad\_Popu=\frac{\Delta f}{\Delta t}=\frac{f\left(p,t_{1}\right)-f\left(p,t_{0}\right)}{t_{1}-t_{0}}$$ (5)

The above formula yields population (density) change with time, which characterizes a general pattern of human mobility in a given area.

**Fig 8:**
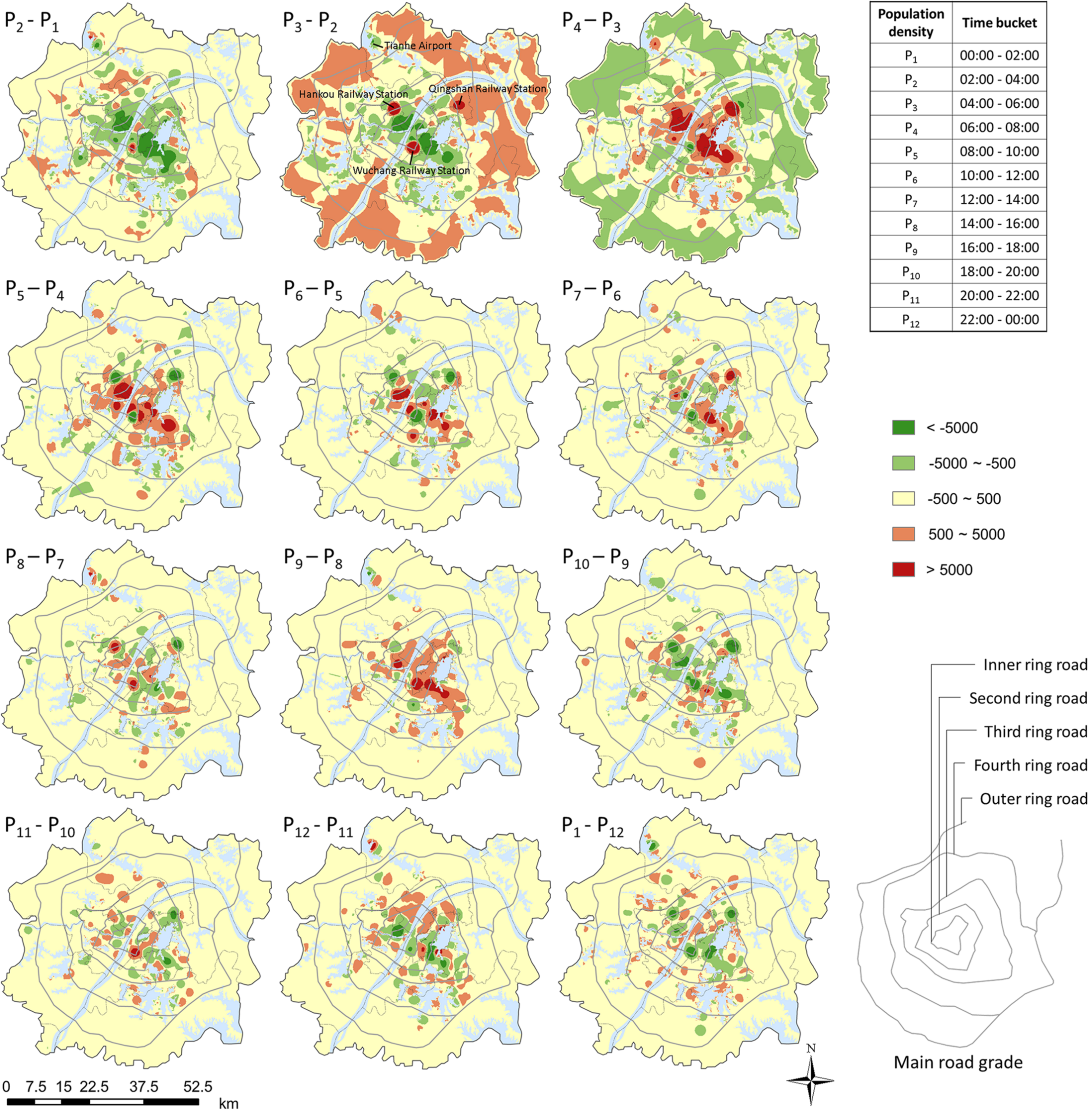
Spatio-temporal distribution of population density gradient.

**Fig 9:**
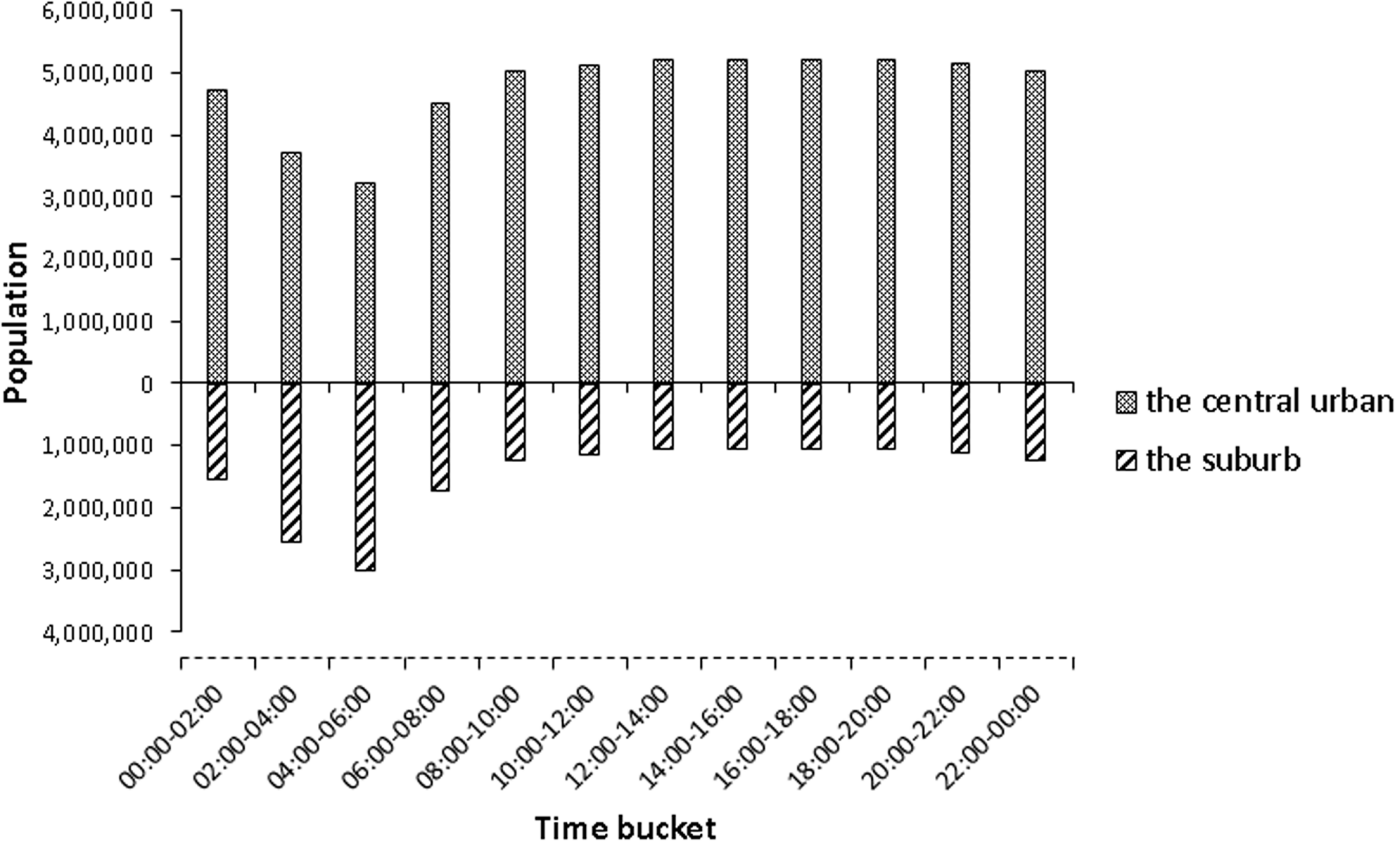
The variation of density in series of time in the suburb and the central urban area.

For the analysis, 12 gradients of population at 12 time-intervals are calculated from the 12 population density sets mentioned above. For simplicity, let time step Δ*t* = 1, then *Grad_Popu* = (*f*(*p*,*t*_1_)−*f*(*p*,*t*_2_)). Fig 8 shows a series of maps of density gradient computed by rasterizing the spatial distribution of population density. Those maps intuitively show the spatio-temporal regularities of human mobility. Fig 9 shows the variation of density in this series of time in the suburb and the central urban area accordingly.

First, upon comparing three gradient maps (P_2_-P_1_, P_3_-P_2_ and P_4_-P_3_) with each other by their color changes in Fig 8 and the height change of histogram at 4 am to 8 am in Fig 9 indicate more activity in the suburb during the period from 4 am to 6 am, as people leave the suburb and move to the central urban area during the period from 6 am to 8 am. An obvious change in density in the urban area and in the suburb presents the general mobility pattern where people move from their residential suburb areas to their work areas in the central urban area. A recognizable though not apparent change in density which could be picked in Fig 9 together with maps P_9_-P_8_ and P_10_-P_9_ in Fig 8 indicates that the movement begins from the window of 4 pm-6 pm from the central urban area to the suburb and it goes steadily until the midnight.

Second, upon comparing gradient changes in the central urban area in three gradient maps (P_2_-P_1_, P_3_-P_2_ and P_4_-P_3_), it is found that three spots (red points) clearly stand out. They are three important transport hubs: railway stations in the city. The gradient change indicates that the three railway stations import and export population from outside of the study area.

Finally, upon further examination of the gradient changes in the maps, it is found that human mobility is very active near commercial centers, and the most representative examples are Jianghan Road and the Simenkou, Jiedaokou and Optics Valley areas. These areas sustain a population density increase from 6 am to 6 pm, and a corresponding decrease in the evening.

## Movement of People Based on WSDE

### Weighted standard deviation ellipse

The standard deviation ellipse (SDE) method [49] is often used to depict spatial characteristics of a geographical entity, such as central tendency, dispersion, and directional trends. SDE not only is an abstract expression for individual spatial activities, but it also builds more comprehensive and realistic models of human mobility [50]. It is quite effective for discrete description of anisotropic events in the spatial point pattern analysis, having been widely used in extensive research such as urban structure analysis [14]. This useful tool is chosen here to analyze the movement of people and travel demands in a more detailed level.

**Fig 10:**
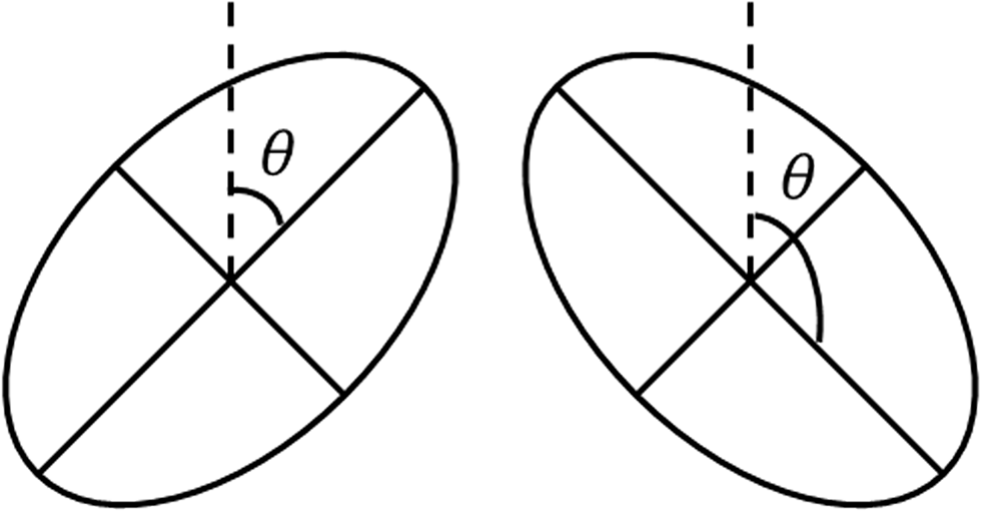
The rotating azimuth of SDE. There are two cases of the rotating azimuth of SDE. In the first case, tan*θ* has a positive value, and in the second case, tan*θ* has a negative value.

The major and minor axes of the SDE are calculated according to Eq 6, and their proportional relations denote the degree of flattening of the SDE. The rotating azimuth is calculated according to Eq 7; as Fig 10 shows, it is the angle between the major axis and due north in a counterclockwise direction. The standard deviations of the major and minor axes of the SDE are calculated according to Eq 8.

$$\begin{array}{l} SDE_{x}=\sqrt{\frac{\sum_{i=1}^{n}{\left(x_{i}-\bar{X}\right)}^{2}}{n}}\\ SDE_{y}=\sqrt{\frac{\sum_{i=1}^{n}{\left(y_{i}-Y\right)}^{2}}{n}} \end{array}\}$$ (6)

In Eq 6, (*x*_*i*_,*y*_*i*_) denotes the coordinates of a check-in point, $\left(\bar{x},\bar{y}\right)$ denotes the geometric center coordinates of point sets, and *n* denotes the number of points.

$$\begin{array}{l} \tan\theta =\frac{A+B}{C}\\ A=\left(\sum_{i=1}^{n}{\tilde{x}}_{i}{}^{2}-\sum_{i=1}^{n}{\tilde{y}}_{i}{}^{2}\right)\\ B=\sqrt{{\left(\sum_{i=1}^{n}{\tilde{x}}_{i}{}^{2}-\sum_{i=1}^{n}{\tilde{y}}_{i}{}^{2}\right)}^{2}+4{\left(\sum_{i=1}^{n}{\tilde{x}}_{i}{\tilde{y}}_{i}\right)}^{2}}\\ C=2\sum_{i=1}^{n}{\tilde{x}}_{i}{\tilde{y}}_{i} \end{array}\}$$ (7)

$$\begin{array}{l} \sigma_{x}=\sqrt{\frac{{\sum_{i=1}^{n}\left({\tilde{x}}_{i}\cos\theta -{\tilde{y}}_{i}\sin\theta \right)}^{2}}{n}}\\ \sigma_{y}=\sqrt{\frac{{\sum_{i=1}^{n}\left({\tilde{x}}_{i}\sin\theta -{\tilde{y}}_{i}\cos\theta \right)}^{2}}{n}} \end{array}\}$$ (8)

In Eqs 7 and 8, ${\tilde{x}}_{i}$ and ${\tilde{y}}_{i}$ denote the deviation between coordinates of an element and the geometric center coordinates of an element set.

To balance the effect caused by the distinction of the occurrence probability, our study, which places population density as a weight on the locations of check-in spots, adopted a weighted standard deviation ellipse (WSDE). The major and minor axes of the WSDE form the spatial region of the population density distribution, and the direction of the major axis is defined as the dominant direction of the variation trend.

### Overall pattern of movement in the case study area shown by WSDE

**Fig 11:**
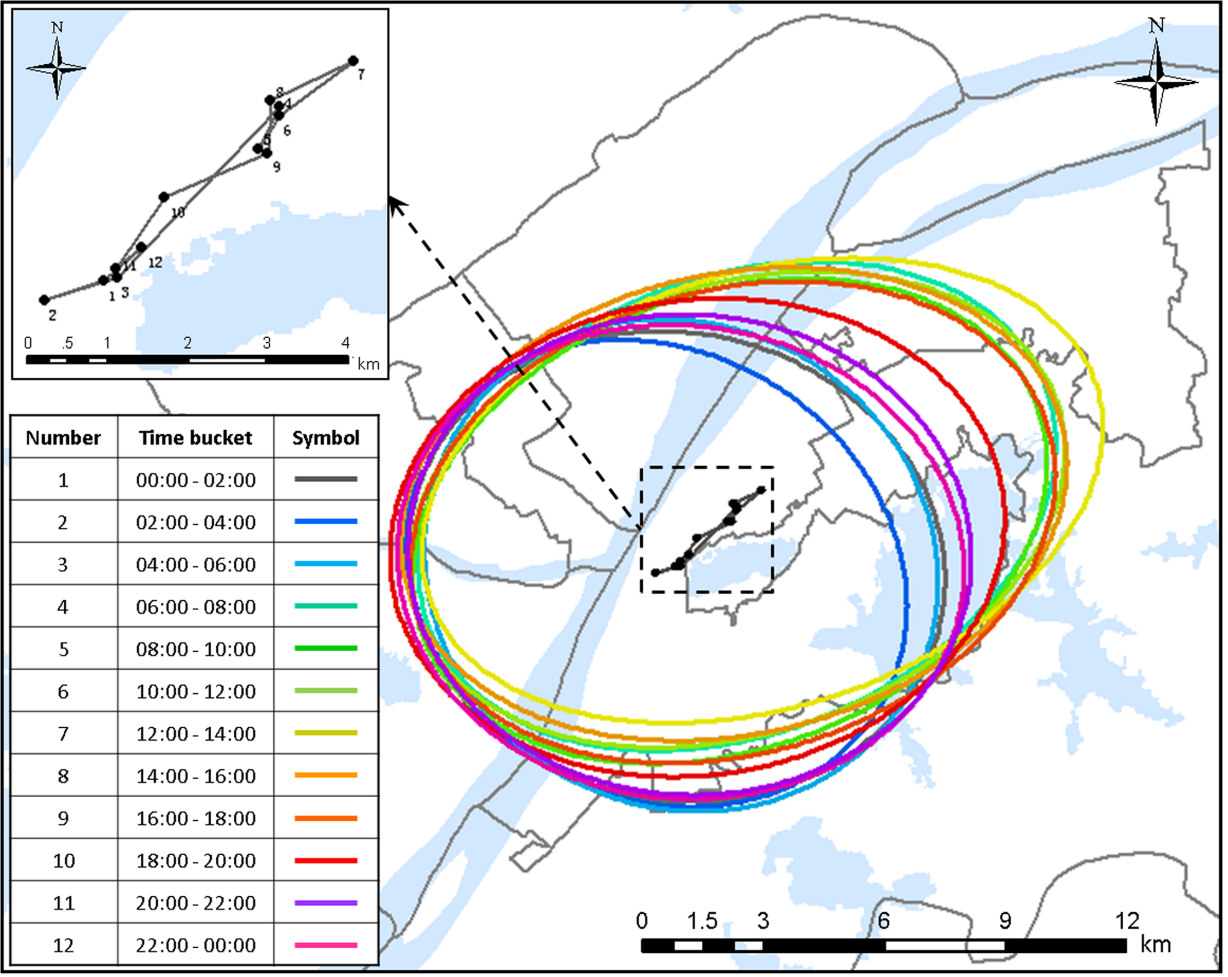
The WSDEs for the transportation travel purpose and the moving trajectories of their centers.

Based on check-in data and density, a series of WSDEs are calculated and plotted in Fig 11, which shows the overall spatial pattern of mobility within the city in terms of public transportation check-in records marked with ‘travel-related’. Its trajectory of the center shows a linear movement in a direction of north-east and south-west, which indicates the major movement of public transportation along with the direction. It closely matches the geographic distribution of the network of transportation in this city because the Yangtze River crosses the city and flows from south-west to north-east. From the WSDEs at different periods, it is known that there is no ellipse coincident with others, the eccentricity of the WSDEs becomes larger and larger from midnight to noon, and the largest eccentricity appears in the period from 12 am to 2 pm. This variation indicates the general extent and intensity of movement in the city by means of transportation as a time series; that is, the inclination to move in the direction of south-west and north-east becomes more and more apparent from equilibrium at midnight, reaching a maximum in the period from 12 am to 2 pm, and after that period the inclination decreases gradually.

### Spatio-temporal characteristics of movement in districts shown in WSDE

**Fig 12:**
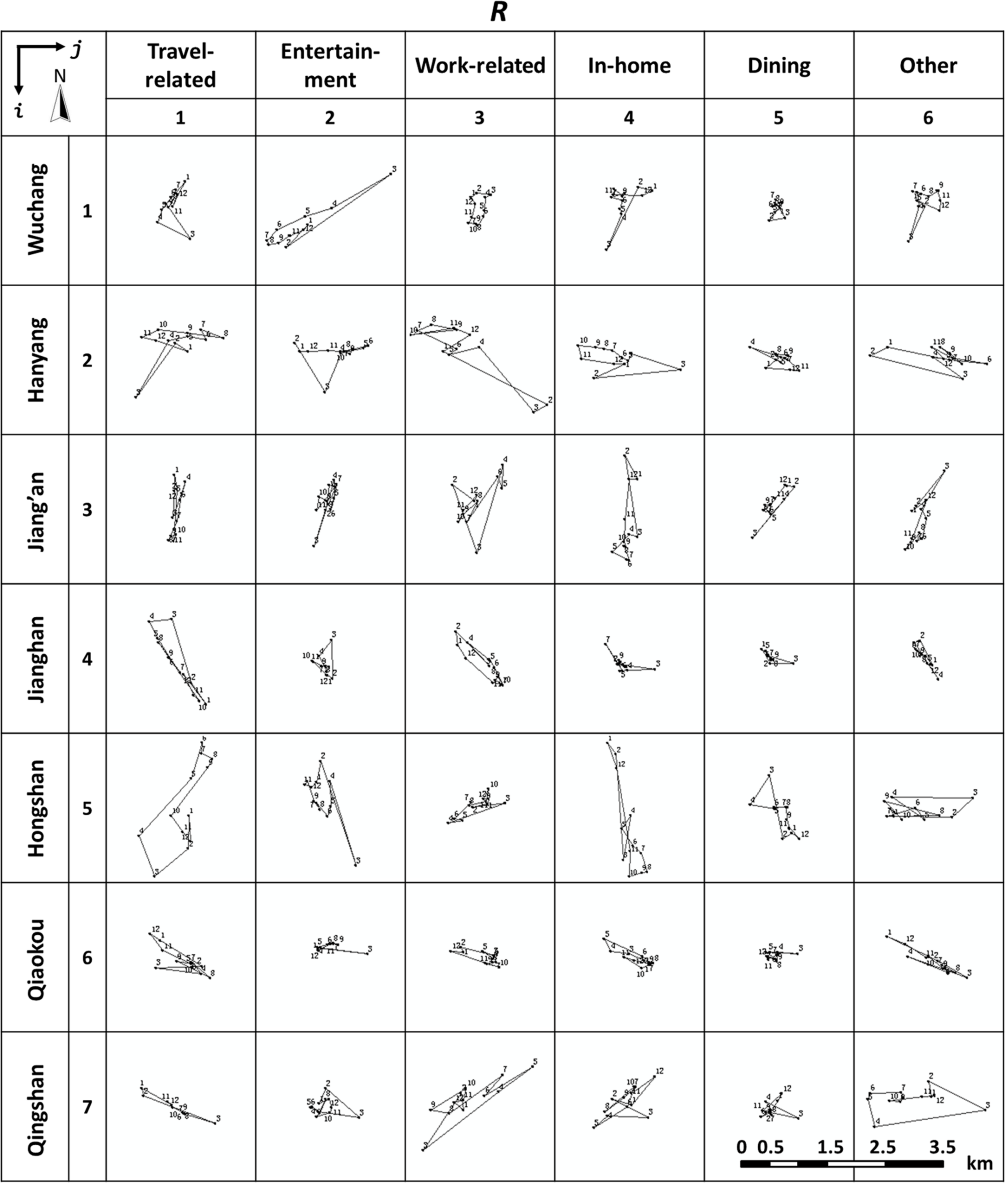
The moving trajectories of the shapes of centers of WSDE.

Furthermore, considering the seven administrative districts in the study area, we plotted the WSDEs for six classes of travel demand for each district. The matrix of district-demand is shown in Fig 12. Each trajectory is treated as an element of the matrix, denoted as *R*_*ij*_, where *i*∈{1,2,…,7} and *j*∈{1,2,…,6}.

The geometry of trajectories indicates different spatial patterns of human mobility in each district in regard to travel demand. In general, the spatial aggregating degree of trajectories in a region indicates the travel distance in that region and a certain degree of temporal autocorrelation on some travel demand. The main reason for choosing 2-hour time window is to reduce data redundancy while keep a certain resolution. We think that the time window with more than 2-hour could not show the enough features of activity in daily life and l-hour or less may be too detailed to present a general and appropriate pattern of human movement. The apparent features of movement are as follows:

1. For all travel demands, the matrix also indicates that a larger movement is required in Hanyang (*R*_2*j*_) and Hongshan (*R*_5*j*_) and a smaller movement occurs in Jianghan (*R*_4*j*_) and Qiaokou (*R*_6*j*_). This may be attributed to Hanyang and Hongshan having a larger area and Jianghan and Qiaokou having a smaller area. The larger area in Hanyang and Hongshan also accounts for the larger extent of the trajectory in *R*_23_, *R*_24_, *R*_26_, *R*_51_, *R*_52_ and *R*_54_ in the matrix, indicating a large movement for certain purposes of travel in a corresponded district. As an example with the demand tagged as ‘travel-related’, the largest extent of the trajectory being *R*_51_ indicates more dispersing in taking public transportation in Hongshan than that in the other districts.
2. Regarding the reason for less travel in a district, if you look for activity for entertainment, Jianghan is the better choice and Wuchang and Hongshan may be the last choices because *R*_42_ has a much smaller extent than *R*_12_ and *R*_52_. If you have work-related tasks in the Wuchang district (*R*_13_), it may take you less effort to commute within that district. However, it is the opposite case if you have to go Hanyang (*R*_23_) or Qingshan (*R*_73_) because *R*_13_ has a much smaller extent of trajectory than *R*_23_ or *R*_73_. Regarding to ‘in-home’, Hanyang(*R*_24_), Jiang’an(*R*_34_) and Hongshan(*R*_54_) have more widespread residential areas than the other regions do. Wuchang, Jianghan and Qiaokou are good regions for gathering for dinner.

It is also found that all travels in two districts have an apparent direction of movement; namely, moving in Jiang’an is likely in an almost north-south direction, and moving in Qiaokou is in an approximately east-west direction. Although check-in data are very raw and imprecise for moving from one destination to another and thus could not to tell the nature of the movement of people among the districts, it is certain from the above analyses that the matrix can provide some spatio-temporal characteristics of human mobility in the urban area, which is very useful for urban planning as well as for daily planning for individuals.

## Conclusions

Check-in data provided by social media networking record the daily activity/travel paths of people in the process of check-in and are an easily-obtainable source of information for the study of intra-urban human mobility. This study has presented an available trial or paradigm for utilizing check-in data with some adopted methods to reveal some characteristics of movement in the urban area. By collecting check-in data for one year and investigating those data, it is possible to provide certain spatio-temporal patterns of the movement of people in the city for some purposes.

Our study suggests that temporal population density in some sense could be approached from the check-in data and be further used for inferring general movement characteristics depending on density changes in the urban area. Its availability could be verified by the consistence of the locations of the peaks of density with the locations of commercial centers in the city. The density changes at a series of times during a day represent a process of movement from the suburb to the central urban area from 4 am to 8 am and dispersing from the central urban area from 4 pm to 8 pm. Three railway stations are identified according to the density changes by the import and export of people in exchange with the outside of the city.

Our analyses by WSDEs indicate that moving directions within the city match well with the principal directions of roads or public transportation lines, and the directions become very clear around noon (from 12 am to 2 pm).

Finally, a series of WSDEs for each district reveal a general moving pattern for certain purposes in each district. The matrix of WSDEs represents the differences in movement patterns in those districts. For example, considering the average moving distance, the larger movement is likely to occur in Hanyang and Hongshan, the smaller movement occurs in Jianghan and Qiaokou, and movement is intermediate in other districts. The matrix can also provide a very useful guide for a person’s daily life. For example, if you want to go for dinner, Wuchang, Jianghan and Qiaokou may be the better choice because the restaurants are fairly compacted in terms of spatial distribution.

Despite the availability and merit of check-in data and the proposed approach in revealing general patterns of intra-urban human mobility, combining check-in data with other more detailed data for movement such as floating car data and other demographic data may reveal more specific moving patterns, which could orient our future study.

## Supporting Information

S1 File Check-in data. (XLSB)

S2 File Check-in user data. (XLSB)
